# Perforated gastric ulcer after unhealthy decisions in a patient with an intragastric balloon, a hangover you will regret

**DOI:** 10.1186/s40792-023-01627-6

**Published:** 2023-03-27

**Authors:** Gabriel A. Molina, Christian Rojas, William Aguayo, Cecilia Vivar, Jose D. Guzmán

**Affiliations:** 1 Digeslap Center & Universidad San Francisco de Quito (USFQ), Quito, Ecuador; 2 Digeslap Center, Quito, Ecuador

**Keywords:** Intragastric balloon, Perforated ulcer, Gastric ulcer

## Abstract

**Background:**

Perforated gastric ulcers are life-threatening surgical emergencies that need early diagnosis and treatment to overcome severe complications. With the rise of obesity in recent years, intragastric balloons have arisen as a "safe" strategy; however, in medicine, no treatment is risk-free. Nausea, pain, vomiting, and more severe complications like perforation, ulceration, and death can occur.

**Case presentation:**

We present the case of a 28-year-old man with obesity; treatment with an intragastric balloon was initiated with good results at the beginning of his treatment. However, he neglected his treatment over time and made unhealthy choices, leading to a severe complication. However, thanks to prompt surgical treatment, he made a full recovery.

Complications:

Gastric perforation following an intragastric balloon is a severe and potentially life-threatening complication that an experienced multidisciplinary team must treat promptly and, more importantly, prevent.

## Introduction

Intragastric balloons (IGB) are temporary, reversible endoluminal devices that treat obesity to improve preoperative conditions and reduce life-threatening comorbidities [1]. Despite their extensive use, they can be associated with rare but life-threatening conditions requiring urgent treatment [1, 2]. We present a case of gastric perforation in a patient with an intragastric balloon in which poor follow-up and unhealthy decisions combined to lead to a complicated scenario. Fortunately, the patient recovered completely.

## Case report

Patient is an otherwise healthy 28-year-old male. He struggled with his weight since his teenage years and tried several weight loss strategies, including diet and exercise, without any long-term success. Therefore, he sought other therapies. After an initial evaluation with a multidisciplinary team that included nutritional and psychological counseling, he accepted treatment with an IGB (A spherical 13 cm silicone-made balloon with a volume of 500 ml was placed) as his body mass index (BMI) was 27 kg/m^2^ at that time.

Four months later, he had great success; he had lost almost 13 kg, reaching a BMI of 24 kg/m^2^. In addition, he changed his habits, exercised more, and adjusted his diet. Nonetheless, he had to endure a very stressful situation as he had to graduate from his university; at this time, he stopped taking omeprazole, started smoking, eating poorly, and started drinking alcohol almost daily to cope with this stress. Then, five months after the IGB, the patient consumed vast amounts of alcohol to the point of intoxication; during this episode, the patient experienced severe abdominal pain, nausea, and vomits. Suddenly the patient started vomiting blood to the point where he became unresponsive and was brought immediately by his family to the emergency room.

On clinical examination, a tachycardic (130 beats/min) and hypotensive patient (Mean arterial pressure: 55 mmHg) was encountered; he had an arterial blood Ph of 7.22 with a lactate of 5.5 mmol/L. Therefore, aggressive intravenous resuscitation was given until the patient's pressure was normal and he regained some consciousness. At this time, severe abdominal pain with tenderness in his upper abdomen was discovered, so complimentary exams were requested.

**Fig. 1:**
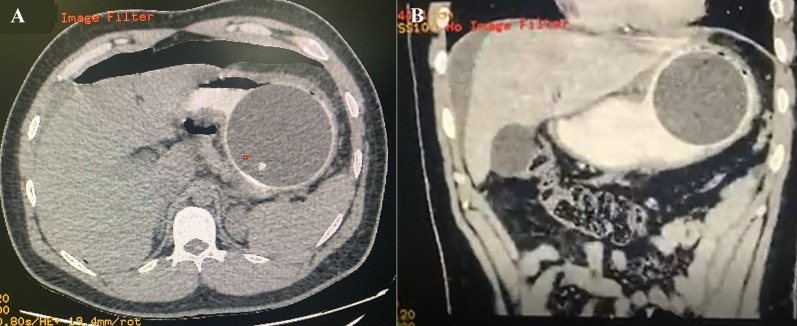
**A** CT, Free air in the abdomen. **B** CT, Perihepatic liquid along with the intragastric balloon

**Fig. 2:**
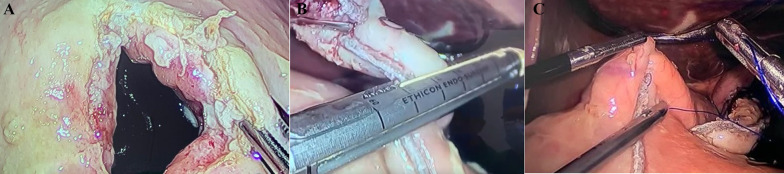
**A** Gastric ulcer in the anterior wall of the stomach. **B** Mechanical Staplers removing the ulcer and closing the gastrostomy. **C** Staple line reinforcement

**Fig. 3:**
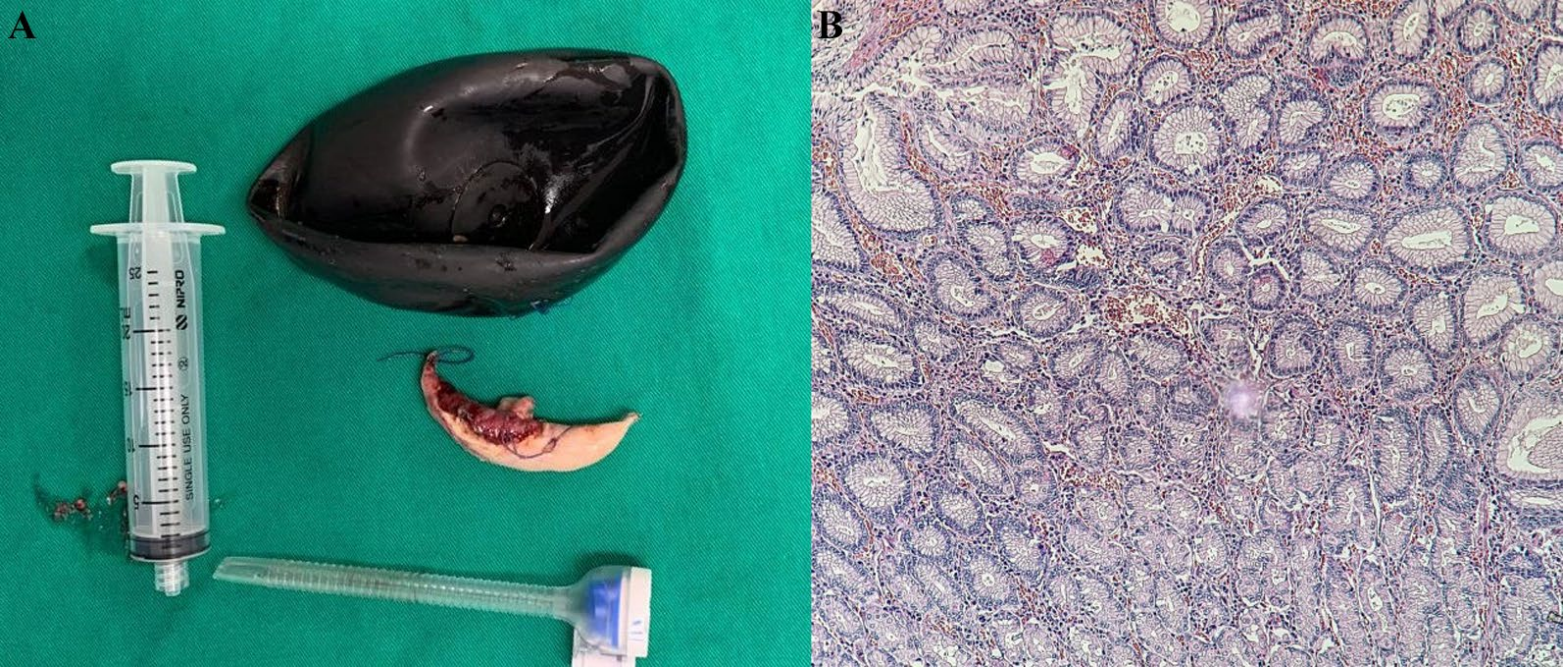
**A** Gastric balloon and ulcer are removed. **B** Pathology, Inflammation with necrosis, and cellular debris was found

A complete blood count (CBC) reported leukocytosis over 12,500 mm^3^ with a neutrophilia of 85%; due to the abdominal pain, a computed tomography (CT) was requested, revealing free air in the abdomen with perihepatic liquid. In addition, the stomach wall was thickened, and the IGB was found on the inside (Fig. 1A, B). With these findings, surgical consultation was needed, and after obtaining consent, surgery was required. On laparoscopy, 300 mL of purulent fluid was found in the abdomen. After thorough revision, 5 cm away from the pylorus, a 1 × 0.7 cm perforated ulcer was discovered on the anterior wall of the stomach (Fig. 2A). With these findings, a 3 cm gastrostomy was done through the perforation. Afterward, the gastric balloon was visualized, deflated, and removed from the stomach. Then, after hemostasis at the ulcer site was achieved, two sutures were placed 2 cm away from the ulcer perforation for traction, and two mechanical staplers (Ethicon Inc., Somerville, New Jersey, USA) were used to remove the ulcer and close the gastrostomy. After this, a staple line reinforcement was done with a non-absorbable suture, the abdominal cavity was cleaned with over 6 L of saline, two drains were placed, and the gastric balloon and the partial gastrectomy were removed (Figs. 2B, C). Pathology confirmed a 1 × 1 cm transmural ulcer surrounded by necrotic tissue with abundant neutrophils and necrosis. The surgical edges are viable (Fig. 3A, B).

The patient's postoperative course was uneventful. Broad-spectrum antibiotics were administered for seven days, the drains were removed on the sixth postoperative day, and the patient was discharged on postoperative day eight without complications. In the follow-ups, the patient is doing very well; he is back to his usual diet and under close supervision from the nutritionist and psychologist.

## Discussion

Peptic ulcer disease is a condition that affects the mucosa of the digestive tract damaging the mucosa and the submucosal layers, which can lead to perforation and sepsis [2]. This disease appears due to the alteration of the protective mucosal lining of the gastrointestinal tract due to dietary factors, an increased acid production, H. pylori infection, NSAID (nonsteroidal anti-inflammatory drugs), and foreign objects, such as an IGB [2, 3]. Other risk factors, such as smoking, have been associated with an increased risk of perforation and alcohol intake with a higher risk of bleeding [3, 4].

The current epidemic of overweight and obesity and its strong links to cardiometabolic diseases, such as type 2 diabetes mellitus, hypertension, and dyslipidemia, has led to the rise of multiple procedures to achieve weight loss, including surgery, endoscopic and metabolic treatments [4, 5]. Nonetheless, many patients will be more inclined to try less invasive procedures mainly because of the fear of postoperative complications [1].

One of these "safe" therapies is IGB, which was first used by Niebeb et al. in 1982 [6]. This type of treatment is based on a space-occupying device built on the principles of a restrictive surgical procedure since they slow down gastric emptying, making patients eat smaller meals and experience satiety for a prolonged time, ultimately leading to weight loss [1, 6]. Currently, there are many kinds of gastric balloons; nonetheless, they all must follow the same principles; they must have a smooth surface with low potential for causing erosions, ulcers, or obstructions; they must be made of durable materials (polyurethane or silicone) that do not leak; they can be filled with liquid or air; must be marked with a radiopaque marker and must have the capability of being adjusted to various sizes [1, 7].

Balloons can have many adverse effects, such as vomiting, abdominal pain, reflux symptoms, esophagitis, and weight regain if no long-term lifestyle changes are adopted [7, 8]. Other severe complications, such as gastric ulceration and perforation, have also been reported but are fortunately rare (0.1%) [1, 7]. These complications can be categorized depending on the device, the patient, or the doctor, as it was reported by Stavrou et al. in 2019 [9]. Gastric perforation due to an IGB is rare, but it can lead to severe morbidity and mortality when it occurs [10, 11]. Ulcers and gastric erosions may occur if PPIs are not prescribed or not taken [12, 13]. Other situations that may increase the risk of perforation are alcohol, NSAID consumption, tobacco, and food residues impacted between the gastric wall and the balloon, which can generate an ischemia zone, which might lead to perforation [9, 14]. In our case, our patient stopped taking proton pump inhibitors, consumed alcohol, and smoked, which led to gastric perforation.

The advertising of easy use and safe therapy can become a disastrous trap if the patient or the multidisciplinary team fails; education and strict supervision of these patients are mandatory to prevent such dangerous complications.

## Conclusion

Gastric perforation following an intragastric balloon is a serious and potentially life-threatening complication that an experienced multidisciplinary team must manage. Since the development of peritoneal complications could be lethal, prompt diagnosis is vital. This case demonstrates that close follow-up is essential when dealing with an obese patient. In addition, prevention of complications through nutritional counseling and replacing unhealthy habits are of utmost importance.

## Data Availability

All our images are our own, and the data can be shared to the Editor on request.
